# The influences of patient's trust in medical service and attitude towards health policy on patient's overall satisfaction with medical service and sub satisfaction in China

**DOI:** 10.1186/1471-2458-11-472

**Published:** 2011-06-15

**Authors:** Liyang Tang

**Affiliations:** 1 Department of Economics, School of Economics and Management, Tsinghua University, Beijing, China

## Abstract

**Background:**

It is widely accepted that patient generates overall satisfaction with medical service and sub satisfaction on the basis of response to patient's trust in medical service and response to patient's attitude towards health policy in China. This study aimed to investigate the correlations between patient's trust in medical service/patient's attitude towards health policy and patient's overall satisfaction with medical service/sub satisfaction in current medical experience and find inspiration for future reform of China's health delivery system on improving patient's overall satisfaction with medical service and sub satisfaction in considering patient's trust in medical service and patient's attitude towards health policy.

**Methods:**

This study collaborated with the National Bureau of Statistics to collect a sample of 3,424 residents from 17 provinces and municipalities in a 2008 China household survey on patient's trust in medical service, patient's attitude towards health policy, patient's overall satisfaction and sub satisfaction in current medical experience.

**Results:**

Patient's overall satisfaction with medical service and most kinds of sub satisfaction in current medical experience were significantly influenced by both patient's trust in medical service and patient's attitude towards health policy; among all kinds of sub satisfaction in current medical experience, patient's trust in medical service/patient's attitude towards health policy had the largest influence on patient's satisfaction with medical costs, the influences of patient's trust in medical service/patient's attitude towards health policy on patient's satisfaction with doctor-patient interaction and satisfaction with treatment process were at medium-level, patient's trust in medical service/patient's attitude towards health policy had the smallest influence on patient's satisfaction with medical facilities and hospital environment, while patient's satisfaction with waiting time in hospital was not influenced by patient's trust in medical service/patient's attitude towards health policy.

**Conclusion:**

In order to improve patient's overall satisfaction with medical service and sub satisfaction in considering patient's trust in medical service and patient's attitude towards health policy, both improving patient's interpersonal trust in medical service from individual's own medical experience/public trust in medical service and improving patient's attitude towards health policy were indirect but effective ways.

## Background

Over the past few decades, patient satisfaction has taken a prominent position in the medical service research literature [1-3]. This attention has been justified since patient satisfaction (directly or indirectly) has become a key criterion for evaluating the quality of medical service and the encounters between medical professional and patient [4-6]. In fact patient satisfaction reflects not only patients' judgment and assessment of their medical experience but also their perception of the gap between what they want and what they receive [7]. Patient satisfaction is a summarizing response that results from patients' post-treatment cognitive and affective evaluation of medical service performance given pre-treatment expectation [8,9].

The central role of trust in medical relationships has been recognized for a long time [10-13]. Trust is seen as a global attribute of treatment relationships, one that encompasses subsidiary features such as satisfaction, communication, competency, and privacy-each of which has considerable importance in its own right [14]. Trust in medical service can be seen as trust in physician and medical institution, and it focuses on two questions "whether the physician and medical institution are competent to make a diagnosis and provide treatment" and "whether the physician and medical institution will act in the best interest of the patient" [15]. Most patients must depend on the physician and medical institution for the information they need to answer these two questions, which results in an unbalanced relationship [15]. The formation process of patient's trust in medical service is the learning and combining process of interpersonal trust in medical service from her/his own medical experience and public trust in medical service [16], then when patient forms stationary trust in medical service as response to the unbalanced relationship between patient and physician/medical institution, it influences patient's attitude towards, cognition on, and response mode with the effectiveness of medical service provided by physician and medical institution to a large extent [17].

Patient's attitude towards health policy also has taken a prominent position in China's medical service research literature [17,18] due to the fact that China's administrative health delivery system (simply denoted as CHDS) was mainly regulated by relevant government agencies including the health administrative agencies and the health insurance agencies, and health policy had strong guidance for the development of CHDS [18]. And evidence from interview in China showed that among patients who received the same medical service, patients who thought highly (lowly) of health policy in recent years usually had higher (lower) satisfaction with medical service in current medical experience [18].

Patient's overall satisfaction with medical service in current medical experience is an aggregation of sub satisfaction involving satisfaction with doctor-patient interaction, satisfaction with treatment process, satisfaction with waiting time in hospital (Most Chinese have no idea of appointment, so waiting time in China mainly refers to waiting time between registration and diagnosis.), satisfaction with medical facilities and hospital environment, and satisfaction with medical costs [17]. It is widely accepted that patients generate multi-attribute-based responses on their overall satisfaction and sub satisfaction [19] in current medical experience [20-23]: the attribute-level responses of overall satisfaction and sub satisfaction are composed of several parts; one major part are responses to severity of disease, stage of disease, treatment effect, medical expense, and reimbursement percentage of medical costs in current medical experience which have significant direct impacts on patient's overall satisfaction and sub satisfaction in current medical experience [17]; the other major part includes not only response to patient's trust in medical service which is a combination of response to interpersonal trust in medical service from her/his own medical experience and response to public trust in medical service [16] but also response to patient's attitude towards health policy, and both of them have indirect impact on patient's overall satisfaction and sub satisfaction in current medical experience [17,23]; a patient is expected to aggregate the attribute-level responses of satisfaction to an overall reflection of satisfaction. This aggregation process is presumed to be a heuristics-based decision-making process. Basically, a patient processes information to come to a decision on whether and to what extend she/he is satisfied with medical service [18]. So in order to improve patient's overall satisfaction and sub satisfaction, one indirect but effective way is to improve patient's trust in medical service (either improve interpersonal trust in medical service from individual's own medical experience or improve public trust in medical service) and patient's attitude towards health policy [17].

To overcome the most important persistent problem "medical service is expensive and difficult to access" in CHDS, which was especially serious in higher-level hospital (mainly refer to level-2 hospital and level-3 hospital. According to the Chinese Ministry of Health's most recent "Governing Rules for the Management and Classification of Hospitals" in 1989, public hospitals in China are classified into three levels: level 1 hospitals are "community hospitals or health clinics that provide direct prevention, treatment, health promotion, and rehabilitation services to participants of a defined community"; level 2 hospitals are "area hospitals that provide comprehensive medical and other healthcare services to participants of multiple communities, which may, to a certain degree, also serve as teaching hospitals and research bases"; level 3 hospitals are those that "provide high-quality, specialty medical and other healthcare services to participants in a minimum of several areas, and also serve as high-level teaching hospitals and conduct sophisticated research".), the Chinese government has already focused on how to improve patient satisfaction with medical service: in 2005 the Ministry of Health began a nationwide hospital management review in order to push health delivery organizations to improve patient satisfaction with medical service; some local governments used patient satisfaction with medical service to evaluate hospital performance and determine financial investment for hospital [24]; many hospitals established patient satisfaction feedback mechanism to improve their medical service quality [17].

The central and local governments have increased financial investments in CHDS for a long time, especially in higher-level hospital. Although the physical capacity of hospital (especially higher-level hospital) has improved quickly along with the increasing investment in buildings, equipments, and medical facilities, the scarce resources, especially human resources, have been misallocated due to improper pricing mechanism of medical service which can't rightly evaluate the advanced and sparse medical services, and although the number of medical professional has increased, the percentage of excellent medical professional has decreased, facing with the sharply increasing scale of patients most medical professionals have still kept on treating disease with outdated technology, as the result the overall quality and reliability of medical service have decreased [23]. Evidences from literature [17,23] all supported that the development of hospital's competence, especially competence of human resources (mainly refers to doctor's limited attention per patient), was usually far behind the increasing of patient's demand, which not only induced serious imbalance between the expansion and the inadequate competence of CHDS, but also induced serious imbalance between patient's increasing demand and CHDS's limited medical service supply. As the result of the two major imbalances, the decreasing quality and reliability of medical service in recent years has induced the decreasing of interpersonal trust in medical service from individual's own medical experience in recent years [23]; due to the persistent imbalance between the public high expectation of medical service and the poor actual effect of medical service provided by CHDS, the public trust in medical service has kept on decreasing in recent years [17,25]; the poor effect of China's health policy which was far below the public expectation has induced the deterioration of public attitude towards health policy in recent years [18]; patient's sub satisfaction with human resource related hospital competence (mainly refer to satisfaction with doctor-patient interaction and satisfaction with treatment quality) were usually lower than patient's sub satisfaction with medical facilities and hospital environment [23]; the persistent problem of long waiting time in hospital was in fact the problem of limited medical service supply and limited competence of CHDS as the result of scarce and inequitably allocated medical resources [26-28], and patients generally had low satisfaction with waiting time in hospital; the most important persistent problem "medical service is expensive and difficult to access" and the problem "supporting hospitals through drug sales" in CHDS have become more and more serious and attracted most public attention in recent years [25], patient's medical costs have kept on increasing and patient's satisfaction with medical costs has kept on decreasing up till now [17].

On the basis of current situation of CHDS, this study attempted to test whether patient's trust in medical service and patient's attitude towards health policy had significant influences on patient's overall satisfaction with medical service and sub satisfaction in current medical experience using a sample of 3,424 residents collected in a 2008 China household survey from 17 provinces and municipalities by the National Bureau of Statistics, and inspiration for future reform of CHDS on improving patient's overall satisfaction with medical service and sub satisfaction in considering patient's trust in medical service and patient's attitude towards health policy was also found.

## Methods

### Data

To obtain data on patient's trust in medical service, patient's attitude towards health policy, patient's overall satisfaction and sub satisfaction in current medical experience, this study collaborated with the National Bureau of Statistics to collect a sample of 3,424 residents from 17 provinces and municipalities in a 2008 China household survey. In this survey both residents and investigators were very patient, and the National Bureau of Statistics approved that the quality of data was high. 93.76% of residents in this survey were urban residents, and only 6.24% of residents in this survey were rural residents, so the analysis in this paper mainly focused on urban residents. The questionnaire consisted of three parts: the first part inquired about patient's overall satisfaction with medical service and sub satisfaction (including satisfaction with doctor-patient interaction, satisfaction with treatment process, satisfaction with waiting time in hospital, satisfaction with medical facilities and hospital environment, and satisfaction with medical costs) in current medical experience; the second part inquired about patient's trust in medical service and patient's attitude towards health policy; the third part inquired about personal information about current medical experience which had significant influence on patient satisfaction in current medical experience, here severity of disease, stage of disease, treatment effect, medical expense, and reimbursement percentage of medical costs were included. The use of the dataset was approved by the National Bureau of Statistics.

The characteristics of the study population in current medical experience were shown in table 1. The population distribution on each characteristic in current medical experience followed the natural distribution in China, which was the result of stratified sampling design by the National Bureau of Statistics. Descriptions for measures of patient's trust in medical service, measures of patient's attitude towards health policy, measures of overall satisfaction and sub satisfaction in current medical experience were in additional file 1.

**Table 1:** Descriptive statistics of sample characteristics in current medical experience

		Total	
		
		Frequency	Percentage
	Significant improvement	2019	58.97%
	Small improvement	1229	35.89%
Treatment effect	No improvement	147	4.29%
	Small deterioration	17	0.50%
	Significant deterioration	12	0.35%

	Total	3424	100.00%

	Not serious	975	28.48%
Severity of disease	General	1770	51.69%
	Serious	531	15.51%
	Unknown	148	4.32%

	Total	3424	100.00%

	Emergency and serious disease	539	15.74%
Stage of the disease	Non-emergency disease at initial stage	2026	59.17%
	Non-emergency disease at medium stage	526	15.36%
	Non-emergency stable disease at late stage	333	9.73%

	Total	3424	100.00%

	[0,108)	856	25.00%
Medical expense (¥)	[108,258)	856	25.00%
	[258,800)	856	25.00%
	[800-	856	25.00%

	Total	3424	100.00%

	100%	238	6.95%
	(70%, 100%)	591	17.26%
Reimbursement percentage of medical costs	[40%, 70%)	500	14.60%
	[20%, 40%)	201	5.87%
	(0%, 20%)	106	3.10%
	0%	1788	52.22%

	Total	3424	100.00%

### Regression model

Ordered probit model is a generalization of the popular probit analysis for the case of more than two outcomes of an ordinal-dependent variable. Since the latent evaluation score y_i _is a linear function of independent variables written as a vector x_i_, here i is sample number, and y_i _= x_i_*b+ε_i_, where b is a vector of coefficients andε_i _is assumed to follow a standard normal distribution. For an ordered probit model with k cutoff points, define p_j _(j = 1,2,...,k) as the cutoff points of all y_i_, then y_i_≦p_1_, p_j_<y_i_≦p_j+1 _(j = 1,2,...,k-1) or y_i_>p_k_. Following the notation [29], the ordered probit model is expressed as(1)(2)(3)

Where y_j _(j = 0,1,...k) is the discrete value of y_i _and _Ф _is the cumulative standard normal distribution function.

The marginal effect of x_i _on the probability of binary can be calculated according to this formula [29]:(4)(5)(6)

Whereφis the standard normal density function, and based on (4), (5) and (6) the vector of coefficient b can be estimated.

### Theoretical model specification

The following ordered probit models (n = 1,2,...,6) were estimated to test the different correlations between patient's trust in medical service/patient's attitude towards health policy and patient's overall satisfaction with medical service/sub satisfaction in current medical experience:(7)

Here y_ni _(n = 1,2,...,6) were patient's overall satisfaction with medical service/sub satisfaction in current medical experience, specifically, y_1i _was patient's overall satisfaction with medical service in current medical experience, y_2i _was patient's satisfaction with doctor-patient interaction in current medical experience, y_3i _was patient's satisfaction with treatment process in current medical experience, y_4i _was patient's satisfaction with waiting time in hospital in current medical experience, y_5i _was patient's satisfaction with medical facilities and hospital environment in current medical experience, and y_6i _was patient's satisfaction with medical costs in current medical experience; x_1i _was patient's trust in medical service, and x_2i _was patient's attitude towards health policy; z_m _was control variable, since individual's satisfaction in current medical experience were influenced by severity of disease, stage of disease, treatment effect, medical expense, and reimbursement percentage of medical costs in current medical experience, they were all controlled as dummy variables in the regression models; error termε_i _was assumed to be distributed normal.

## Results

The descriptive statistics of patient's trust in medical service, patient's attitude towards health policy, and patient's overall satisfaction with medical service/sub satisfaction in current medical experience were shown in table 2.

**Table 2:** Descriptive statistics of patient's trust, attitude and satisfaction

	Number of observations	Mean	Standard deviation	Min	Max
Patient's trust in medical service	3424	2.79	1.33	1	5
Patient's attitude towards health policy	3424	3.55	0.74	1	5
Overall satisfaction with medical service	3424	3.68	0.76	1	5
Satisfaction with doctor-patient interaction	3424	3.60	0.75	1	5
Satisfaction with treatment process	3424	3.51	0.77	1	5
Satisfaction with waiting time in hospital	3424	3.22	1.04	1	5
Satisfaction with medical facilities and hospital environment	3424	3.37	0.68	1	5
Satisfaction with medical costs	3424	3.44	0.80	1	5

Regression results of theoretical models were in table 3. From results of regression model 1-6, among patient's overall satisfaction with medical service and all kinds of sub satisfaction, the coefficients for both patient's trust in medical service x_1i _(p < 0.05) and patient's attitude towards health policy x_2i _(p < 0.01) in regression model 1-3, 5, 6 were all significantly positive, while only the coefficients for both patient's trust in medical service x_1i _(p > 0.1) and patient's attitude towards health policy x_2i _(p > 0.1) in regression model 4 were not significant, which revealed that patients who had higher degree of trust in medical service or who had more optimistic attitude towards health policy usually not only had higher overall satisfaction with medical service in current medical experience, but also had higher satisfaction with doctor-patient interaction, higher satisfaction with treatment process, higher satisfaction with medical facilities and hospital environment, and higher satisfaction with medical costs in current medical experience, while patient's satisfaction with waiting time in hospital in current medical experience wasn't influenced by either patient's trust in medical service or patient's attitude towards health policy.

**Table 3:** Regression results of theoretical models

	(1)	(2)	(3)	(4)	(5)	(6)
	
	Overall satisfaction with medical service	Satisfaction with doctor-patient interaction	Satisfaction with treatment process	Satisfaction with waiting time in hospital	Satisfaction with medical facilities and hospital environment	Satisfaction with medical costs
Patient's trust in medical service	0.757***	0.439**	0.413***	-0.0780	0.0474***	0.610**
	(5.05)	(2.39)	(2.91)	(-0.74)	(3.27)	(2.28)
Patient's attitude towards health policy	0.319***	0.201***	0.214***	0.123	0.0246***	0.242***
	(9.41)	(6.24)	(6.68)	(0.43)	(7.45)	(6.07)

Severity of disease dummy	Yes	Yes	Yes	Yes	Yes	Yes
Stage of disease dummy	Yes	Yes	Yes	Yes	Yes	Yes
Treatment effect dummy	Yes	Yes	Yes	Yes	Yes	Yes
Medical expense dummy	Yes	Yes	Yes	Yes	Yes	Yes
Reimbursement percentage of medical costs dummy	Yes	Yes	Yes	Yes	Yes	Yes

Cutoff point 1	-1.407***	-1.143***	-1.383***	-1.552***	-1.980***	-1.102***
	(-3.76)	(-3.25)	(-3.92)	(-4.62)	(-5.54)	(-2.67)
Cutoff point 2	-0.332	-0.692**	-0.940***	-0.580*	-1.077***	-0.330
	(-0.90)	(-1.98)	(-2.68)	(-1.73)	(-3.08)	(-0.80)
Cutoff point 3	0.317	1.171***	0.839**	0.537	0.931***	1.289***
	(0.85)	(3.35)	(2.39)	(1.60)	(2.66)	(3.14)
Cutoff point 4	2.621***	2.451***	2.121***	1.306***	2.240***	2.499***
	(7.03)	(6.99)	(6.02)	(3.90)	(6.37)	(6.07)

Number of observations	3424	3424	3424	3424	3424	3424
Log pseudo-likelihood	152.6	92.37	103.2	79.9	83.1	109.19

*t *statistics in parentheses, * *p *< 0.10, ** *p *< 0.05, *** *p *< 0.01

From the sizes of the significant coefficients for both patient's trust in medical service x_1i _and patient's attitude towards health policy x_2i _in each regression model, generally speaking, among all kinds of sub satisfaction, patient's trust in medical service/patient's attitude towards health policy had the largest influence on patient's satisfaction with medical costs, the influences of patient's trust in medical service/patient's attitude towards health policy on patient's satisfaction with doctor-patient interaction and satisfaction with treatment process were at medium-level, while patient's trust in medical service/patient's attitude towards health policy had the smallest influence on patient's satisfaction with medical facilities and hospital environment. Since patient's overall satisfaction with medical service was an aggregation of all kinds of sub satisfaction in current medical experience, the influence of patient's trust in medical service/patient's attitude towards health policy on patient's overall satisfaction with medical service was larger than the influence of patient's trust in medical service/patient's attitude towards health policy on any kind of sub satisfaction.

## Discussion

Patient's overall satisfaction with medical service and most kinds of sub satisfaction in current medical experience were significantly influenced by patient's trust in medical service/patient's attitude towards health policy; the influence of patient's trust in medical service/patient's attitude towards health policy on patient's overall satisfaction with medical service could be considered as an aggregation of the influences of patient's trust in medical service/patient's attitude towards health policy on all kinds of sub satisfaction in current medical experience; among all kinds of sub satisfaction in current medical experience, patient's trust in medical service/patient's attitude towards health policy had the largest influence on patient's satisfaction with medical costs due to the fact that the persistent problem "medical service is expensive and difficult to access" in China was the most important problem patients paid most attention to in considering their trust in medical service/attitude towards health policy in their medical experiences [17,25]; the influences of patient's trust in medical service/patient's attitude towards health policy on patient's satisfaction with doctor-patient interaction and satisfaction with treatment process were larger than the influence of patient's trust in medical service/patient's attitude towards health policy on patient's satisfaction with medical facilities and hospital environment, this was because human resources, especially the number and quality of senior medical professional in CHDS had not increased concurrently to keep pace with the excessive physical expansion (mainly refer to expansion of buildings, equipments, and medical facilities in hospitals) of CHDS, then in considering trust in medical service and attitude towards health policy patients usually paid more attention to human resource related hospital competence (mainly refer to doctor-patient interaction and treatment quality) than medical facilities and hospital environment [23,25]; the reason why patient's trust in medical service/patient's attitude towards health policy had no significant influence on patient's satisfaction with waiting time in hospital in current medical experience was that long waiting time was a general problem in CHDS and patient's trust in medical service/patient's attitude towards health policy was no longer important in patient's consideration of satisfaction with waiting time in hospital [17].

On the basis of these findings, this study found the following inspiration for future reform of CHDS on improving patient's overall satisfaction with medical service and sub satisfaction in considering patient's trust in medical service and patient's attitude towards health policy: the increase of patient's trust in medical service and the improvement of patient's attitude towards health policy could induce the increases of patient's overall satisfaction with medical service and most kinds of sub satisfaction, the Chinese government could focus on improving patient's trust in medical service (involving improving patient's interpersonal trust in medical service from individual's own medical experience and improving public trust in medical service) and improving patient's attitude towards health policy to indirectly but effectively improve patient's overall satisfaction with medical service and sub satisfaction; in order to increase patient's interpersonal trust in medical service from individual's own medical experience, the most effective way was to improve the quality and reliability of medical service; the practical way for the Chinese government to increase the public trust in medical service was to solve the persistent imbalance between the public high expectation of medical service and the poor actual effect of medical service provided by CHDS; in order to improve patient's attitude towards health policy, more practical health policy on improving medical service should be formulated and put in place for the public; the Chinese government should not only emphasis on financial investment in physical capacity of CHDS, in fact human resources, especially the number and quality of senior medical professional in higher-level hospital have not increased concurrently to keep pace with either the excessive physical expansion of CHDS or patient's increasing demand for medical service, so the development of human resources in CHDS (especially in higher-level hospital) should be paid much more attention to in order to improve patient's satisfaction with doctor-patient interaction and satisfaction with treatment process in considering patient's trust in medical service and patient's attitude towards health policy; the Chinese government should also release inappropriate administrative intervention for CHDS, use proper and efficient management to inspire hospital (especially higher-level hospital) to supply advanced medical services at proper price to patient in need in order to improve patient's satisfaction with medical costs in considering patient's trust in medical service and patient's attitude towards health policy, and further alleviate the persistent problem "medical service is expensive and difficult to access" [23]; the problem of long waiting time in hospital was fundamentally caused by limited medical service supply and limited competence of CHDS, only when medical resources were more abundant and were more equitably allocated [26,27,30-32] can patient's satisfaction with waiting time in hospital be improved in considering patient's trust in medical service and patient's attitude towards health policy.

## Conclusion

This study found that both patient's trust in medical service and patient's attitude towards health policy had significant influences of different levels on patient's overall satisfaction with medical service and most kinds of sub satisfaction (involving satisfaction with doctor-patient interaction, satisfaction with treatment process, satisfaction with medical facilities and hospital environment, and satisfaction with medical costs) in current medical experience. And this study found the following inspiration for future reform of CHDS on improving patient's overall satisfaction with medical service and sub satisfaction in considering patient's trust in medical service and patient's attitude towards health policy: patient's interpersonal trust in medical service from individual's own medical experience, public trust in medical service, and patient's attitude towards health policy should be improved in order to indirectly but effectively improve patient's overall satisfaction with medical service and sub satisfaction; the quality and reliability of medical service should be improved; the persistent imbalance between the public high expectation of medical service and the poor actual effect of medical service provided by CHDS should be solved; more practical health policy on improving medical service should be formulated and put in place for the public; inappropriate administrative intervention for CHDS should be released; the development of human resources in CHDS should be paid much more attention to; medical resources should be made more abundant and allocated more equitably.

## Competing interests

The author declares that they have no competing interests.

## Authors' contributions

LT designed the study, performed the statistical analysis, interpreted the results and drafted the manuscript.

## Pre-publication history

The pre-publication history for this paper can be accessed here:

http://www.biomedcentral.com/1471-2458/11/472/prepub

## Supplementary Material

Additional file 1 **Measures of patient's trust in medical service, measures of patient's attitude towards health policy, measures of overall satisfaction and sub satisfaction in current medical experience**. Click here for file
