# Supplementary material for: The influences of patient's trust in medical service and attitude towards health policy on patient's overall satisfaction with medical service and sub satisfaction in China
Source: BMC Public Health. 2011 Jun 15;11:472. doi: 10.1186/1471-2458-11-472 (PMC3129314; doi:10.1186/1471-2458-11-472)
Supplement: Additional file 1 — Measures of patient's trust in medical service, measures of patient's attitude towards health policy, measures of overall satisfaction and sub satisfaction in current medical experience. [file 1471-2458-11-472-S1.DOC]

**Measures of patient’s trust in medical service**

The 5-item self-reporting measure that accessed patient’s trust in medical service on a scale of 1 to 5 was employed, higher score reflected higher degree of trust in medical service: the option “Have high degree of trust in medical service and always follow instruction from medical service” was assigned score 5; the option “Have relatively high degree of trust in medical service and follow instruction from medical service most of the time” was assigned score 4; the option “Have medium degree of trust in medical service and follow instruction from medical service sometimes” was assigned score 3; the option “Have relatively low degree of trust in medical service and don’t follow instruction from medical service most of the time” was assigned score 2; and the option “Have low degree of trust in medical service and always choose not to follow instruction from medical service” was assigned score 1.

**Measures of patient’s attitude towards health policy**

A 5-item self-reporting measure that accessed patient’s attitude towards health policy on a scale of 1 to 5 was employed, higher score reflected more optimistic attitude towards health policy in recent years: the option “China’s health policy in recent years had significant improvement” was assigned score 5; and the option “China’s health policy in recent years had small improvement” was assigned score 4; the option “China’s health policy in recent years had no improvement” was assigned score 3; the option “China’s health policy in recent years had small deterioration” was assigned score 2; and the option “China’s health policy in recent years had great deterioration” was assigned score 1.

**Measures of overall satisfaction and sub satisfaction in current medical experience**

The 5-item self-reporting measures that accessed patient’s overall satisfaction with medical service and sub satisfaction (including satisfaction with doctor-patient interaction, satisfaction with treatment process, satisfaction with waiting time in hospital, satisfaction with medical facilities and hospital environment, and satisfaction with medical costs) in current medical experience on a scale of 1 to 5 were employed, higher scores reflected higher overall satisfaction with medical service/higher sub satisfaction in current medical experience: the option “Very satisfied” was assigned score 5; the option “Quite satisfied” was assigned score 4; and the option “Basically satisfied” was assigned score 3; the option “Quite dissatisfied” was assigned score 2; and the option “Very dissatisfied” was assigned score 1.
